# Isolation and Identification of Quercetin Degrading Bacteria from Human Fecal Microbes

**DOI:** 10.1371/journal.pone.0090531

**Published:** 2014-03-04

**Authors:** Zhichao Zhang, Xichun Peng, Shaoting Li, Ning Zhang, Yong wang, Hua Wei

**Affiliations:** 1 Department of Food Science and Engineering, Jinan University, Guangzhou, China; 2 State Key Laboratory of Food Science and Technology, Nanchang University, Nanchang, China; Institut Pasteur of Shanghai, Chinese Academy of Sciences, China

## Abstract

Quercetin has a wide range of biological properties. The gut microflora can often modulate its biological activity and their potential health effects. There still is a lack of information about gut bacteria involving in this process. The strains of gut microbes from human feces that can transform quercetin were isolated and identified by *in vitro* fermentation. The results showed that *Escherichia coli*, *Stretococcus lutetiensis*, *Lactobacillus acidophilus*, *Weissella confusa*, *Enterococcus gilvus*, *Clostridium perfringens* and *Bacteroides fragilis* have the various ability of degrading quercetin. Among them, *C. perfringens* and *B. fragilis* were discovered to have the strongest ability of degrading quercetin. Additionally, quercetin can't inhibit the growth of *C. perfringens*. In conclusion, many species of gut microbiota can degrade quercetin, but their ability are different.

## Introduction

Quercetin (3, 3′, 4′, 5, 7-pentahydroxyflavone) is a flavonoid widely distributed in plant foods and herbal medicines. It has a wide range of biological properties including anti-oxidization [1], [2], anti-mutagenicity [3], anti-inflammatory effects [4] and the prevention or decrease of cardiovascular diseases [5]. These beneficial effects could be related to the native forms of quercetin present in vegetables and fresh fruit, or their metabolites.

In general, the absorption of polyphenols in the digestive tract starts in the ileum, where the more complex structures (esters, glycosides, or polymers) that cannot be absorbed in their native form are hydrolysed by intestinal enzymes or the colonic microflora [6]. The non-absorbed polyphenols in the ileum will reach the colon whole [7]–[9], and be transformed by the gut microbiota enzymes (esterase, glucosidase, demethylation, dehydroxylation and decarboxylation enzymatic activities) [10] into a wide range of low-molecular-weight phenolic acids [11]. For example, polyphenols can be transformed by colonic microflora into phenolic acids, such as phenylvaleric, phenylpropionic, phenylacetic, benzoic and hippuric acids [12]. Thus, the gut microflora can often modulate the biological activity of these dietary polyphenols [13], [14], and their potential health effects by the degrading process.

Although it is known that the intestinal microflora participate in the metabolism of flavonoids [15], there is a lack of information about the species/strains that may involved in the process. The aim of this research was to isolate and identify the bacterial strains in the human gut that can transform quercetin (one of flavonoids) in an *in vitro* model. Furthermore, the fermentation properties of the isolated strain(s) with quercetin were evaluated.

## Materials and Methods

### 2.1. Fecal sample collection and selective bacterial culture

Fecal bacteria were isolated from freshly collected human feces under anaerobic conditions by diluting the feces with saline peptone water (0.1% (w/v) peptone and 0.85% (w/v) NaCl in distilled water). 100 ul of fecal suspension were then plated on the various selective agar media with 1% quercetin (Qiyun Biological Science & Technology Limited Co.), These selective media (Qingdao Hope Biol-Technology Co, Ltd) included Bile Esculin Azide Agar (BEA), MRS Agar and Brain Heart Infusion Broth (BHI) with 1.5% agar. The selective plates were individually incubated at 37°C in different incubator for 16 h, such as BEA in aerobic incubator, MRS in an anaerobic CO_2_ incubator inflated with air containing 5% CO_2_ and BHI agar in an anaerobic incubator.

### 2.2. Isolation of strains

All different colonies were picked up from each plate according to their appearance of. All isolates were purified by streak plate method and then stored at −80°C in a freezing medium [16] until used.

### 2.3. Verification of strains

The ability of transforming quercetin of the isolates was further verified with the following procedure: the strains were individually inoculated into their corresponding broth with 1% quercetin for 16 h. Then the broths were centrifuged at 5000 rpm for 3 min and the absorbance of supernatant was measured under 415 nm with UV/VIS Spectrophotometer after reacted 10 min with equal volume solution of 2% AlCl_3_ dissolved in methanol. The broth with quercetin but no strains was set as the negative control. All tests were parallelled three times.

### 2.4. Extraction and preparation of genomic DNA from isolates

Isolates were individually cultured for 12 h in 1.5 ml corresponding selective broth at 37°C and cells were individually collected by centrifugation at 5000 rpm for 3 min. Broth was removed -and genomic DNA was extracted from the cell pellets by Invitrogen™ genomic DNA extraction kits. The extracted genomic DNA from each isolate was checked by horizontal gel electrophoresis with 0.8% (w/v) agarose containing 0.5 µg/ml ethidium bromide in 0.5X TBE. The gel was visualized using an Image Master® VDS (Amersham Plc., Buckinghamshire, UK). The DNA concentration and purity were also determined using a spectrophotometric method (UV/VIS spectrophotometer, Shimadzu Corp., Kyoto, Japan) described by Maniatis et al. [17]. The DNA preparations were stored in 40 µl nuclease-free water at −20°C.

### 2.5. Amplification of 16S rDNA of isolates

The 16S rDNA fragment of each isolate was amplified by PCR with the universal primer set of 27-f (5′-AGT TTG ATC CTG GCT CAG-3 ′) and 1492-r (5′-GTT ACC TTG TTA CGA CTT C-3′) according to Naomi et al. [18] using an iCycler Thermal Cycler (Bio-Rad Laboratories Inc., Hercules, USA). All reagents used in PCR amplification were purchased from Fermentas International Inc., Ontario, Canada. The amplification was done in 50 µl reaction volumes as described by Hoefel et al. [19]. Each PCR reaction consisted of 200 µM of each dNTP, 1.0 µM of each primer, 2.5 mM MgCl_2_, 1X PCR buffer, 2.5 U of Taq DNA polymerase, and 50 ng DNA template. The thermal cycling included an initial denaturation step at 95°C for 10 min; 30 cycles of a denaturation step at 95°C for 30 s, an annealing step at 50°C for 1 min, an extension step at 72°C for 2 min; and a final extension at 72°C for 10 min. The PCR product was checked using 0.8% (w/v) agarose gel electrophoresis. The gel was visualized using an Image Master® VDS.

### 2.6. 16S rDNA sequencing

DNA sequence analysis was performed by Shanghai Sanggong Inc., Shanghai, China. Homology searches of the 16S rDNA sequences were performed in the GenBank with the Blast program.

### 2.7. Proof and quantitative test of identified strains metabolizing quercetin

All identified strains were further proved by the chromogenic reaction: The cells were centrifuged from their corresponding broth medium and sprayed with the methanol solution of 2% AlCl_3_. If the cells can transform quercetin into other substances, they show grayish white (their own color); and if quercetin is not transformed by the bacteria, it would be absorbed at the surface of cells and react with aluminium chloride, then present yellow. Then the remaining of quercetin in supernatant broth was colorimetrically tested and compared by the method described above (see 2.3).

### 2.8 Statistics

Results are expressed as mean values with their standard deviation (SD). Statistical analyses were conducted with the Statistical Package for Social Science (SPSS for Windows, version 8.0; SPSS Inc., Chicago, IL, USA) to determine if variables differed among treatment groups.

## Results

### 3.1. Strains transforming quercetin

All isolates were picked up from three kinds of media plate and the counts were present at Table 1. By the reduction of absorbance, 8 strains from BEA were verified to have the ability of metabolizing quercetin, 30 from MRS and 8 from BHI (Table 1).

**Table 1:** Isolates, verified and identified strains from three selective media.

Media	No. of Isolates	No. of Verified strains by 16S rDNA sequencing	Closest Neighbors of Similarity
BEA	24	8	*Enterococcus gilvus*
MRS	30	6	*Lactobacillus acidophilus*
		9	*Weissella confuse*
		11	*Stretococcus lutetiensis*
		4	*Escherichia coli*
BHI	41	2	*Bacteroides fragilis*
		6	*Clostridium perfringens*

Abbreviations: BEA-Bile Esculin Azide Agar, MRS-medium invented by de Man, Rogosa and Sharpe, BHI-Brain Heart Infusion Broth.

### 3.2. Molecular identification by 16S rDNA

**Figure 1:**
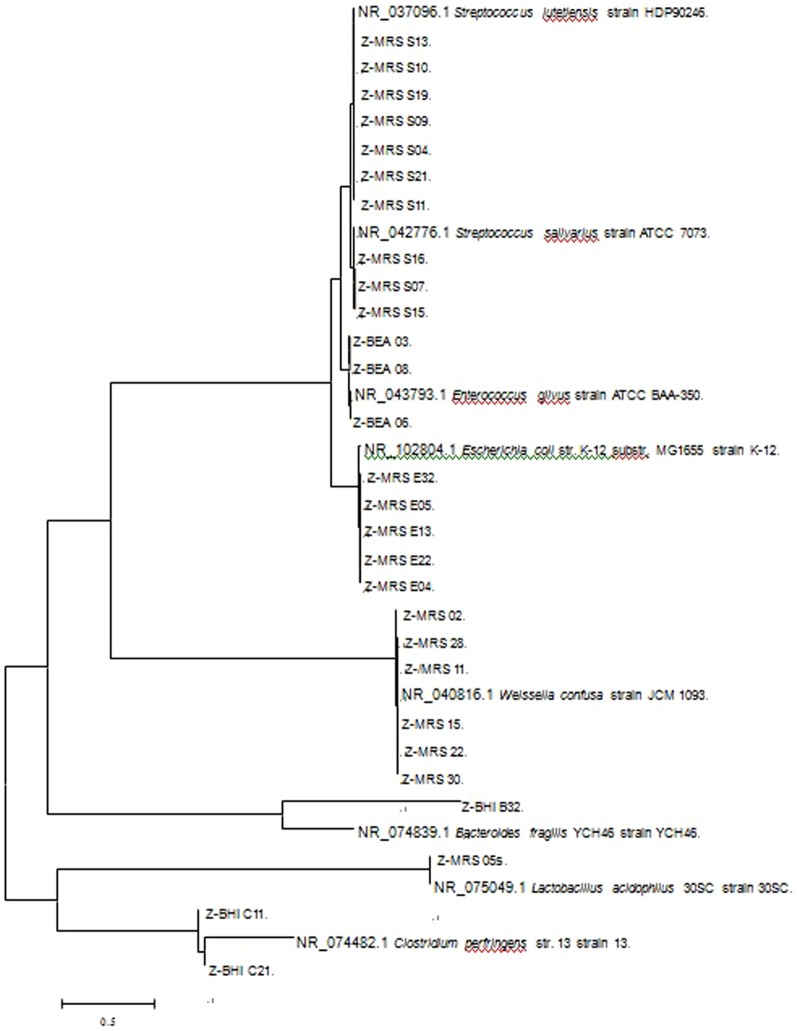
The phylogenetic tree of strains transforming quercetin.

The above strains were further identified by 16S rDNA sequencing and showed in Table 1. Eight strains from BEA plates were verified were allas *Enterococcus gilvus* (99% similarity); there were four species including *Lactobacillus acidophilus*, *Weissella confusa*, *Stretococcus lutetiensis* and *Escherichia coli* (>97% similarity)identified in the MRS plates - a medium mainly used to selectively cultivate *Lactobacillus* spp; there were two species identified in BHI plates, *Bacteriodes fragilis* and *Clostridium perfringens*. The phylogenetic tree of these strains was present in Figure 1.

### 3.3. Proof and comparison of strains identified in metabolizing quercetin

**Figure 2:**
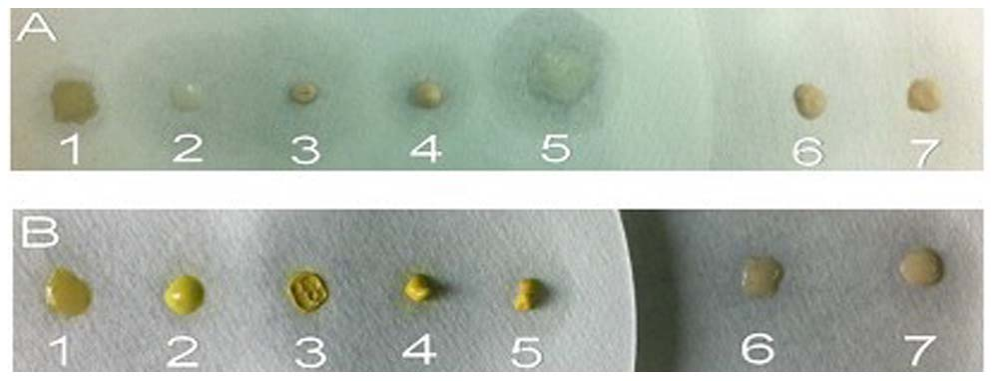
Different cells centrifuged from broths without quercetin (A) or with quercetin (B). All cells were sprayed with 2% AlCl_3_ methanol solution to develop color. The dots of 1–7 refer to *Escherichia coli*, *Stretococcus lutetiensis*, *Lactobacillus acidophilus*, *Weissella confusa*, *Enterococcus gilvus*, *Clostridium perfringens* and *Bacteroides fragilis*.

In the chromogenic reaction, the strains of *E. coli*, *S. lutetiensis*, *L. acidophilus*, *W. confuse* and *E. gilvus* present more yellow (Figure 2-B) than their corresponding controls (Figure 2-A). While the color of *B. fragilis* and *C. perfringens* showed grey which were similar to their corresponding control (Figure 2-A). The results indicated that some quercetin had been absorbed in the surface of strains 1–5, but not in the surface of 6–7.

**Figure 3:**
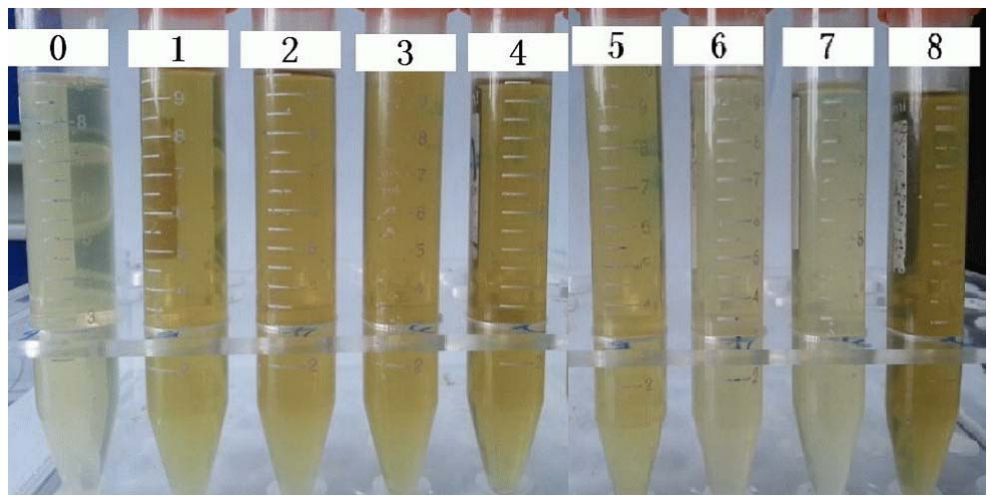
The supernatant broths with quercetin after fermented by different bacteria. The tubes of 1–7 individually refer to the supernatant broths inoculated with *Escherichia coli*, *Stretococcus lutetiensis*, *Lactobacillus acidophilus*, *Weissella confusa*, *Enterococcus gilvus*, *Clostridium perfringens* and *Bacteroides fragilis*. Tube 0 is the positive control without any strains and quercetin; tube 8 is the negative control with quercetin but no any strains.

**Figure 4:**
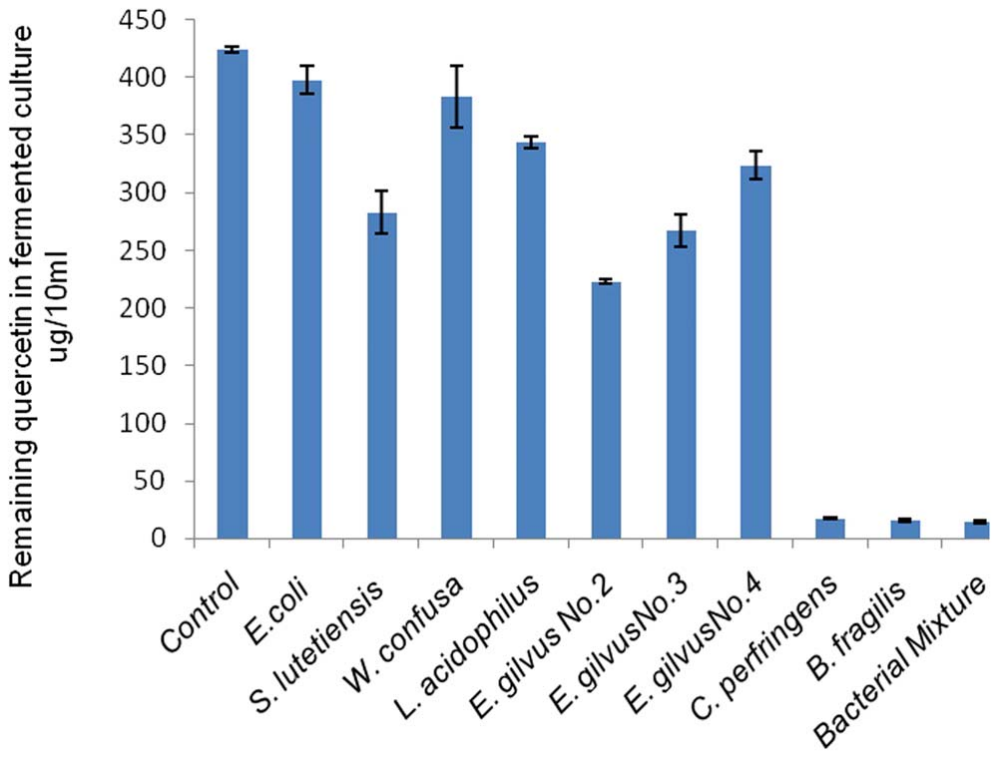
The remains of quercetin fermented by different bacteria in broths. The control refers to the broth with quercetin but no bacterial strain.

The comparison of different bacteria transforming quercetin was further present in Figure 3 and Figure 4. Only a little of quercetin was metabolized by *E. coli*, *S. lutetiensis*, *L. acidophilus*, *W. confuse* and *E. gilvus* due to their broth color changed only a little compared to the negative control (Figure 3, Tube 8); however, the broth of *B. fragilis* and *C. perfringens* present no color like the positive control (Tube 1). According to the quantitative test, *B. fragilis* and *C. perfringens* transformed 95.99% and 96.27%, respectively remaining 1.58±0.14 µg/mL and 1.71±0.07 µg/mL quercetin, which almost equals to the degrading rate (96.66%) of bacterial mixture (1.42±0.07 µg of remaining quercetin). But *E. coli*, and *W. confusa* only broke down a little quercetin (6.19% and 9.60%) after 24 h fermentation, remaining 39.79±1.16 µg/mL quercetin. *L. acidophilus* and *S. lutetiensis* also presented their capacity in degrading quercetin (19.06% and 33.33%, respectively). The different strains of *E. gilvus* also present different capacity of metabolizing quercetin (47.45%, 37.03% and 23.67%) (Figure 4).

## Discussion

Many literatures had already reported gut microbiota with the capacity of metabolizing flavonoids [19]–[24]. The strains that have hitherto been reported to degrade quercetin included *Eubacterium oxidoreducens* [19], *Clostridium orbiscindens* [21], *E. ramulus* [23] and the other four undescribed *Clostridium* sp. [20].

In this paper, seven different bacteria that can transform quercetin were isolated from fresh human feces, including *E. gilvus*, *S. lutetiensis*, *E. coli*, *L. acidophilus*, *W. confusa*, *C. perfringens* and *B. fragilis*. The difference of metabolizing quercetin among these strains has also been presented in this paper. Among them, *B. fragilis* and *C. perfringens* have the highest capacity. However, the *B. fragilis* group is the least common *Bacteroides* present in fecal flora, comprising only 0.5% of the bacteria present in stool [25]. *C. perfringens* (formerly known as *C. welchii*), considered to be relative with several gut chronic diseases, is also less in health gut [26], [27]. Hence, quercetin can't be totally degraded by the two species for their minor amount in human gut. Figure 4 showed that quercetin had been almost totally broken down by the fecal mixture bacteria, which might be attributed to some other strains which were not isolated in current study or to the broth suitable for the growth of the two species.

As reported, plant polyphenols exert significant effects on the intestinal environment by modulation of the intestinal bacterial population, probably by acting as metabolic prebiotics. The growth of certain pathogenic bacteria such as *C. perfringens*, *C. difficile* and *Bacteroides* spp. were significantly repressed by plant phenolics and their derivatives, while commensal anaerobes like *Clostridium* spp., *Bifidobacterium* spp. and probiotics such as *Lactobacillus* spp. were less severely affected [15]. However, *C. perfringens* were firstly verified to highly degrade quercetin in this paper, hence quercetin can't inhibit the growth of *C. perfringens*. *B. fragilis* was also discovered the ability of degrading quercetin as *C. perfringens*, and their ability of degrading quercetin is stronger than the others discovered in this paper.

This study screened the degrading ability of human gut bacteria on quercetin, and discovered that many species of gut microbiota can degrade quercetin, but their ability are different. Two new species with totally degrading quercetin were isolated and identified.

## Conclusion

*E. gilvus*, *S. lutetiensis*, *E. coli*, *L. acidophilus*, *W. confusa*, *C. perfringens* and *B. fragilis* were first discovered to have the ability of degrading quercetin in this paper. And their degrading ability was different. Among them, *C. perfringens* and *B. fragilis* have the strongest ability of degrading quercetin. Quercetin can't inhibit the growth of *C. perfringens*.

## References

[1] Yoshino S, Hara A, Sakakibara H, Kawabata K, Tokumura A, et al (2011) Effect of quercetin and glucuronide metabolites on the monoamine oxidase-A reaction in mouse brain mitochondria. Nutrition 27: 847–852. 21371861 10.1016/j.nut.2010.09.002

[2] Azuma K, Ippoushi K, Terao J (2010) Evaluation of tolerable levels of dietary quercetin for exerting its antioxidative effect in high cholesterol-fed rats. Food Chem Toxicol 48: 1117–1122. 20138950 10.1016/j.fct.2010.02.005

[3] Chang Y, Lin H, Chan S, Yeh S (2012) Effects of quercetin metabolites on the enhancing effect of b-carotene on DNA damage and cytochrome P1A1/2 expression in benzo [a] pyrene-exposed A549 cells. Food Chem 133: 445–450. 25683418 10.1016/j.foodchem.2012.01.060

[4] Lotito SB, Zhang W, Yang CS, Crozier A, Frei B (2011) Metabolic conversion of dietary flavonoids alters their anti-inflammatory and antioxidant properties. Free Radical Biol Med 51: 454–463. 21571063 10.1016/j.freeradbiomed.2011.04.032 PMC3119369

[5] Ishizawa K, Yoshizumi M, Kawai Y, Terao J, Kihira Y, et al (2011) Pharmacology in health food: metabolism of quercetin in vivo and its protective effect against arteriosclerosis. J Pharmacol Sci 115: 466–470. 21436601 10.1254/jphs.10r38fm

[6] Manach C, Scalbert A, Morand C, Rémésy C, Jiménez L (2004) Polyphenols: Food sources and bioavailability. Am J Clin Nutr 79: 727–747. 15113710 10.1093/ajcn/79.5.727

[7] Manach C, Williamson G, Morand C, Scalbert A, Rémésy C (2005) Bioavailability and bioefficacy of polyphenols in humans. I. Review of 97 bioavailability studies. Am J Clin Nutr 81: 2305–2425. 10.1093/ajcn/81.1.230S 15640486

[8] Rasmussen SE, Frederiksen H, Krogholm KS, Poulsen L (2005) Dietary proanthocyanidins: Occurrence, dietary intake, bioavailability, and protection against cardiovascular disease. Mol Nutr Food Res 49: 159–174. 15635686 10.1002/mnfr.200400082

[9] Walle T (2004) Absorption and metabolism of flavonoids. Free Radical Biol Med 36: 829–837. 15019968 10.1016/j.freeradbiomed.2004.01.002

[10] Zoetendal EG, Akkermans ADL, De Vos WM (1998) Temperature gradient gel electrophoresis analysis of 16S rRNA from human fecal samples reveals stable and host-specific communities of active bacteria. Appl Environ Microbiol 64: 3854–3859. 9758810 10.1128/aem.64.10.3854-3859.1998 PMC106569

[11] Jacobs DM, Gaudier E, van Duynhoven J, Vaughan EE (2009) Non-digestible food ingredients, colonic microbiota and the impact on gut health and immunity: A role for metabolomics. Curr Drug Metab 10: 41–54. 19149512 10.2174/138920009787048383

[12] Serra A, Macia A, Romero M, Reguant J, Ortega N, et al (2012) Metabolic pathways of the colonic metabolism of flavonoids (flavonols, flavones and flavanones) and phenolic acids. Food Chem 130: 383–393.

[13] Setchell KDR, Brown NM, Lydeking-Olsen E (2002) The clinical importance of the metabolite equol – A clue to the effectiveness of soy and its isoflavones. J Nutr 132: 3577–3584. 12468591 10.1093/jn/132.12.3577

[14] Xu X, Harris KS, Wang H, Murphy PA, Hendrich S (1995) Bioavailability of soybean isoflavones depends upon gut microflora in women. J Nutr 125: 2307–2315. 7666247 10.1093/jn/125.9.2307

[15] Rastmanesh R (2011) High polyphenol, low probiotic diet for weight loss because of intestinal microbiota interaction. Chem Biol Interact 189: 1–8. 20955691 10.1016/j.cbi.2010.10.002

[16] Gibson LF, Khoury JT (1986) Storage and survival of bacteria by ultra-freeze. Lett Appl Microbiol 3: 127–129.

[17] Maniatis T, Fritch EF, Sambrook J (1982) Molecular Cloning: A Laboratory Manual, New York: Cold Spring Harbor Laboratory Press

[18] Hoefel D, Monis PT, Grooby WL, andrews S, Saint CP (2005) Profiling bacterial survival through a water treatment process and subsequent distribution system. J Appl Microbiol 99: 175–186. 15960678 10.1111/j.1365-2672.2005.02573.x

[19] Krumholz LR, Bryant MP (1986) *Eubacterium oxidoreducens* sp. nov. requiring H_2_ or formate to degrade gallate, pyrogallol, phloroglucinol and quercetin. Arch Microbiol 144: 8–14.

[20] Winter J, Moore LH, Dowell VR, Bokkenheuser VD (1989) C-ring cleavage of flavonoids by human intestinal bacteria. Appl Environ Microbiol 55: 1203–1208. 2757380 10.1128/aem.55.5.1203-1208.1989 PMC184277

[21] Winter J, Popoff MR, Grimont P, Bokkenheuser VD (1991) *Clostridium orbiscindens* sp. nov., a human intestinal bacterium capable of cleaving the flavonoid C-ring. Int J Syst Bacteriol 41: 355–357. 1883711 10.1099/00207713-41-3-355

[22] Schneider H, Schwiertz A, Collins MD, Blaut M (1999) Anaerobic transformation of quercetin-3-glucoside by bacteria from the human intestinal tract. Arch Microbiol 171: 81–91. 9914304 10.1007/s002030050682

[23] Braune A, Gütschow M, Engst W, Blaut M (2001) Degradation of quercetin and luteolin by Eubacterium ramulus. Appl Environ Microbiol 67: 5558–5567. 11722907 10.1128/AEM.67.12.5558-5567.2001 PMC93344

[24] Lu L, Qian D, Yang J, Jiang S, Guo J, et al (2012) Identification of isoquercitrin metabolites produced by human intestinal bacteria using UPLC-Q-TOF/MS. Biomed Chromatogr 27: 509–514. 23018801 10.1002/bmc.2820

[25] Wexler HM (2007) Bacteroides: the good, the bad, and the nitty-gritty. Clin Microbiol Rev 20: 593–621. 17934076 10.1128/CMR.00008-07 PMC2176045

[26] Tannock GW (2010) The bowel microbiota and inflammatory bowel diseases. Int J Inflam 2010: 1–9. 10.4061/2010/954051 PMC3004003 21188223

[27] Tannock GW, Lawley B, Munro K, Lay C, Taylor C, et al (2012) Comprehensive analysis of the bacterial content of stool from patients with chronic pouchitis, normal pouches, or familial adenomatous polyposis pouches. Inflamm Bowel Dis 18: 925–934. 22114001 10.1002/ibd.21936

